# 5-Lipoxygenase-Dependent Recruitment of Neutrophils and Macrophages by Eotaxin-Stimulated Murine Eosinophils

**DOI:** 10.1155/2014/102160

**Published:** 2014-02-25

**Authors:** Ricardo Alves Luz, Pedro Xavier-Elsas, Bianca de Luca, Daniela Masid-de-Brito, Priscila Soares Cauduro, Luiz Carlos Gondar Arcanjo, Ana Carolina Cordeiro Faria dos Santos, Ivi Cristina Maria de Oliveira, Maria Ignez Capella Gaspar-Elsas

**Affiliations:** ^1^Department of Immunology, IMPG, Universidade Federal do Rio de Janeiro, CCS, Bloco I, Room I-2-066, 21941-590 Rio de Janeiro, Brazil; ^2^Department of Pediatrics, IFF, FIOCRUZ, 22250-020 Rio de Janeiro, Brazil; ^3^Department of Medical Microbiology, IMPG, Universidade Federal do Rio de Janeiro, 21941-590 Rio de Janeiro, Brazil

## Abstract

The roles of eosinophils in antimicrobial defense remain incompletely understood. In ovalbumin-sensitized mice, eosinophils are selectively recruited to the peritoneal cavity by antigen, eotaxin, or leukotriene(LT)B4, a 5-lipoxygenase (5-LO) metabolite. 5-LO blockade prevents responses to both antigen and eotaxin. We examined responses to eotaxin in the absence of sensitization and their dependence on 5-LO. BALB/c or PAS mice and their mutants (5-LO-deficient ALOX; eosinophil-deficient GATA-1) were injected i.p. with eotaxin, eosinophils, or both, and leukocyte accumulation was quantified up to 24 h. Significant recruitment of eosinophils by eotaxin in BALB/c, up to 24 h, was accompanied by much larger numbers of recruited neutrophils and monocytes/macrophages. These effects were abolished by eotaxin neutralization and 5-LO-activating protein inhibitor MK886. In ALOX (but not PAS) mice, eotaxin recruitment was abolished for eosinophils and halved for neutrophils. In GATA-1 mutants, eotaxin recruited neither neutrophils nor macrophages. Transfer of eosinophils cultured from bone-marrow of BALB/c donors, or from ALOX donors, into GATA-1 mutant recipients, i.p., restored eotaxin recruitment of neutrophils and showed that the critical step dependent on 5-LO is the initial recruitment of eosinophils by eotaxin, not the secondary neutrophil accumulation. Eosinophil-dependent recruitment of neutrophils in naive BALB/c mice was associated with increased binding of bacteria.

## 1. Introduction

Eosinophils are a minority granulocyte population, which contributes to the pathophysiology of allergic inflammation, hypereosinophilic syndromes, and some malignancies [1–4]. A role for eosinophils in resistance to *multicellular* (helminth) parasites has long been proposed, based on the strong association of blood and tissue eosinophilia with worm infections and on the evidence that eosinophils can damage or kill helminths, in specific experimental conditions [5, 6]. Nevertheless, a generally protective in vivo role for eosinophils against worm infections remains elusive [4], partly because host responses to multicellular parasites represent a compromise between the competing needs to reduce parasite burden and to limit immune-mediated tissue damage, to which eosinophils significantly contribute [7, 8].

Alternatively, mechanisms through which eosinophils may directly fight infection by various classes of *microbial *(bacterial, fungal, protozoal, or viral) pathogens include secretion of antimicrobial defensin-like proteins [9]; release of sticky cellular contents that capture pathogens, closely resembling neutrophil extracellular traps [10]; secretion of halogen microbicidal derivatives [11]; release of enzymes with antiviral activity and other roles in innate immunity [12, 13]; and secretion of a wide array of immunoregulatory cytokines [14]. While the contribution of eosinophils to immunity as directly antimicrobial effector cells is likely limited by their scarcity, they could be helpful in conditions in which neutrophil access or macrophage function would be reduced: for neutrophils, tissue entry is restricted in normal conditions [15]; regarding macrophages, their microbicidal effector function is highly dependent on appropriate activating signals, including cytokines [16]. By contrast, eosinophils are far more numerous in normal tissues than in blood and home to mucosal interfaces with the environment [2–4], which represent potential gateways for microbial infection. They are a source of numerous immunoregulatory cytokines [13] and lipid mediators [17], which might play a role in recruitment/activation of other leukocyte subtypes.

Because of the scarcity of eosinophils, many important observations were made in conditions in which their numbers are already increased, due to allergic sensitization or experimental helminth infection, such as the discovery of eotaxin (CCL11), a chemoattractant that induces eosinophil accumulation in the skin of sensitized (i.e., eosinophilic) guinea pigs [2, 3, 18]. While other potent eosinophil chemoattractants, such as PGD2 [19, 20] and oxo-ETE [21], have also been characterized, many factors reinforce the current understanding of eotaxin as a specialized chemoattractant which acts primarily on granulocyte subtypes relevant to allergy and worm infections [2–4]. These factors include the reported selectivity of eotaxin for the eosinophil [4, 17, 22, 23] and basophil [20, 24, 25] lineages, and its interaction with hematopoietic cytokines, such as IL-5 [26, 27] and GM-CSF [27], which promotes eosinophil production in bone marrow [27] and extramedullary sites [26], ultimately inducing blood and tissue eosinophilia [4]. The alternative view, namely, that eotaxin is part of a broader regulatory network comprising multiple cell populations in addition to eosinophils and basophils, is also suggested by observations of a wide variety of eotaxin effects, including its ability to attract neutrophils and macrophages [28] and smooth muscle cells [29]. Eotaxin, also produced by fibroblasts [30, 31], has been associated with fibrotic processes in several settings [32, 33].

Within this wider framework, we have reexamined whether, in a nonsensitized host, eotaxin would recruit other leukocyte populations besides eosinophils and basophils and further examined whether its effects were dependent on 5-lipoxygenase (5-LO), the key enzyme in the leukotriene production by eosinophils [17, 27]. The evaluation of both aspects was prompted by observations in mice which develop eosinophilia in response to subcutaneously implanted insoluble antigen pellets [34]. While i.p. challenge of implant recipients with soluble allergen selectively recruited eosinophils to the peritoneal cavity, this effect was blocked by the 5-LO-activating protein inhibitor, MK886, and duplicated by the 5-LO product, LTB4, neither of which is eosinophil-selective. Importantly, eotaxin, which duplicated the effects of allergen, was equally blocked by MK886. Equally unexpected was the failure of LTB4, a potent neutrophil chemoattractant, to recruit neutrophils, while it effectively attracted eosinophils in this allergic model. These observations raised the possibility that the eosinophil selective effect of both chemoattractants (eotaxin and LTB4) observed in vivo was dependent on the host being sensitized. We tested this hypothesis for eotaxin first, by examining its effects in a naïve host, as well as the effect of 5-LO blockade on the effectiveness of eotaxin. We report that eotaxin recruits a mixed leukocyte population to the peritoneal cavity of naïve mice and provide evidence of essential roles for both 5-LO and eosinophils in the accumulation and functional activation of neutrophils in this model.

## 2. Materials and Methods

### 2.1. Reagents

RPMI 1640 medium (SH30011.01) and fetal calf serum (SH30088.03) were from Hyclone (Logan, UT); Penicillin 100 U/mL (PEN-B), Streptomycin 100 mg/mL (S9137), Ovalbumin (grade II and grade IV), isotonic Percoll, and Histopaque density 1.083 solution from Sigma-Aldrich (St. Louis, MO); recombinant murine Eotaxin (250-01) from PeproTech (Rocky Hill, NJ); and recombinant murine IL-5 from R&D; MK-886 (475889) 1 mg/kg from Cayman Chemicals (Ann Arbor, MI), dissolved in 0.1% methylcellulose, was given as an intragastric bolus in a 0.2 mL volume [33]. Rat anti-murine eotaxin monoclonal neutralizing antibody (clone 42285) and rat anti-murine IgG2a control monoclonal antibody of matched isotype (clone 54447) were from R&D (Minneapolis, MN).

### 2.2. Animals and Animal Handling

Inbred mice, male and female, aged 8–10 weeks, provided in SPF condition by CECAL-FIOCRUZ (Rio de Janeiro), were of the following strains: BALB/c; ALOX (5-LO-deficient) and PAS-129 (wild-type control of the same background) [27]; and BALB/c mutants lacking an enhancer element in the promoter region of gene coding for the GATA-1 transcription factor [35], required for eosinophil lineage determination (GATA-1 mice, for short). Animal housing, care, and handling followed institutionally approved (CEUA number L-010/04, CEUA number L-002/09) protocols. Naive animals received eotaxin i.p., in 0.2 mL of RPMI 1640 medium with Penicillin/Streptomycin. Controls received medium (RPMI). After the indicated times, animals were killed in a CO_2_ chamber, and peritoneal lavage was carried out with 10 mL chilled RPMI. For sensitized animals, see Section 2.6.

### 2.3. Neutralization of Eotaxin Activity

50 ng eotaxin was incubated with 5 *μ*g anti-eotaxin neutralizing antibody or 5 *μ*g isotype-matched anti-IgG2a antibody, in a final volume of 200 *μ*L, for 30 minutes, before injection into each BALB/c recipient. 4 h later, peritoneal lavage fluid was collected from the injected mice and handled as detailed above.

### 2.4. Collection, Enumeration, and Staining of Peritoneal Leukocytes

Peritoneal lavage cells were washed at 500 ×g and resuspended in 2 mL RPMI. Total counts were carried out in a hemocytometer after a 1 : 10 dilution in Turk's solution. Differential counts were done on Giemsa-stained (ice-cold methanol-fixed, air-dried, and Giemsa-stained for 5 minutes) cytocentrifuge smears (500 rpm, 8 minutes in a Cytospin 3, Thermo Scientific, Waltham, MA), by counting at least 300 cells in 1000x magnification under oil.

### 2.5. Bacteria and Phagocytosis Assay

We used nonpathogenic *Escherichia coli* bacteria (clone DH5, provided by Dr. Z. Vasconcelos, from INCA and FIOCRUZ, Rio de Janeiro) genetically altered to constitutively express the gene for green fluorescent protein (GFP), grown in LB broth. The cells obtained in the peritoneal lavage of BALB/c mice, induced by eotaxin or RPMI, were subjected to total cell count as well as differential neutrophil counts as previously described. Then 5 × 10^5^ neutrophils were incubated for 30 minutes, in the dark at room temperature, with the bacteria in a 1 : 400 proportion. The cells were then washed and the resulting cell suspension was run in a FACScalibur flow cytometer (Becton Dickinson Immunocytometry Systems, San Jose, CA), with the acquisition of at least 50.000 events, and analyzed with the help of Summit 4.3 software (Dako Cytomation, UK).

### 2.6. Eosinophil Procedures

For eosinophil transfer studies, where indicated, BALB/C, ALOX or PAS mice were sensitized (100 *μ*g ovalbumin grade IV and 1,6 mg alum in a final volume of 400 *μ*L saline per animal, two s. c. injections in the dorsum, at days 0 and 7) and challenged (ovalbumin grade IV, 1 *μ*g in 400 *μ*L saline i.p. at day 14) according to Ebihara and colleagues [36]. Bone marrow was collected 48 h after i.p. challenge, examined, and cultured as previously described elsewhere [37]. Briefly, bone-marrow cultures were established for 5 days at 37°C in 95% air/5% CO2, in RPMI1640, with 10% FBS and 5 ng/mL IL-5, at a culture density of 1 × 10^6^ cells/mL. The nonadherent cells were then collected and loaded on top of 3 mL of a Histopaque-1083 solution, followed by centrifugation at 400 ×g, 20°C, 35 minutes, without brakes. The mononuclear cell ring and the supernatant were discarded; the granulocyte-rich pellet was collected, washed and resuspended in 3 mL RPMI, and used for total and differential counts as above. The suspension contained ≥80% eosinophils, with no neutrophils, and the minor contaminant population consisted of macrophages alone, which do not interfere with the interpretation of transfer experiments. Where indicated, naive GATA-1, ALOX, or PAS recipient mice were injected with 1 × 10^6^ eosinophils from the appropriate donors (see below) i.p., followed by eotaxin 50 ng/mL, and leukocyte accumulation was monitored in the peritoneal lavage fluid 4 h after eotaxin injection, as above.

For flow cytometric studies of CCR3 expression, the following modification of this protocol was adopted, for it yielded eosinophils of higher purity: sensitized mice were challenged twice, initially by aerosol exposure (1 h, Ovalbumin grade II, 2.5%, w/v, at day 14) and 7 h later with soluble ovalbumin i.p. (grade IV, 1 *μ*g in 400 *μ*L saline). Bone marrow was collected 24 h after aerosol challenge and cultured as above, after separation on a Percoll gradient (75%/60%/45% isotonic Percoll, 100 ×g, 20 min, room temperature). The hematopoietic cells from the 45%/60% interface [38] were cultured at a lower IL-5 concentration (2.5 ng/mL) for twice as long (10 days), yielding a population containing at least 95% eosinophils, with mature morphology. Contaminants at day 10 were degenerating (nonviable) mononuclear and stromal cells.

### 2.7. Statistical Analyses

All data were analyzed with Systat for Windows 5.04 (Systat, Inc. Everston, IL, USA), using the two-tailed *t*-test for pairwise comparisons. Where indicated, ANOVA was also used for multiple comparisons, with the Tukey HSD correction and the Bonferroni correction for groups of equal and unequal size, respectively.

## 3. Results

### 3.1. Mixed Leukocyte Migration Induced by Eotaxin

**Figure 1:**
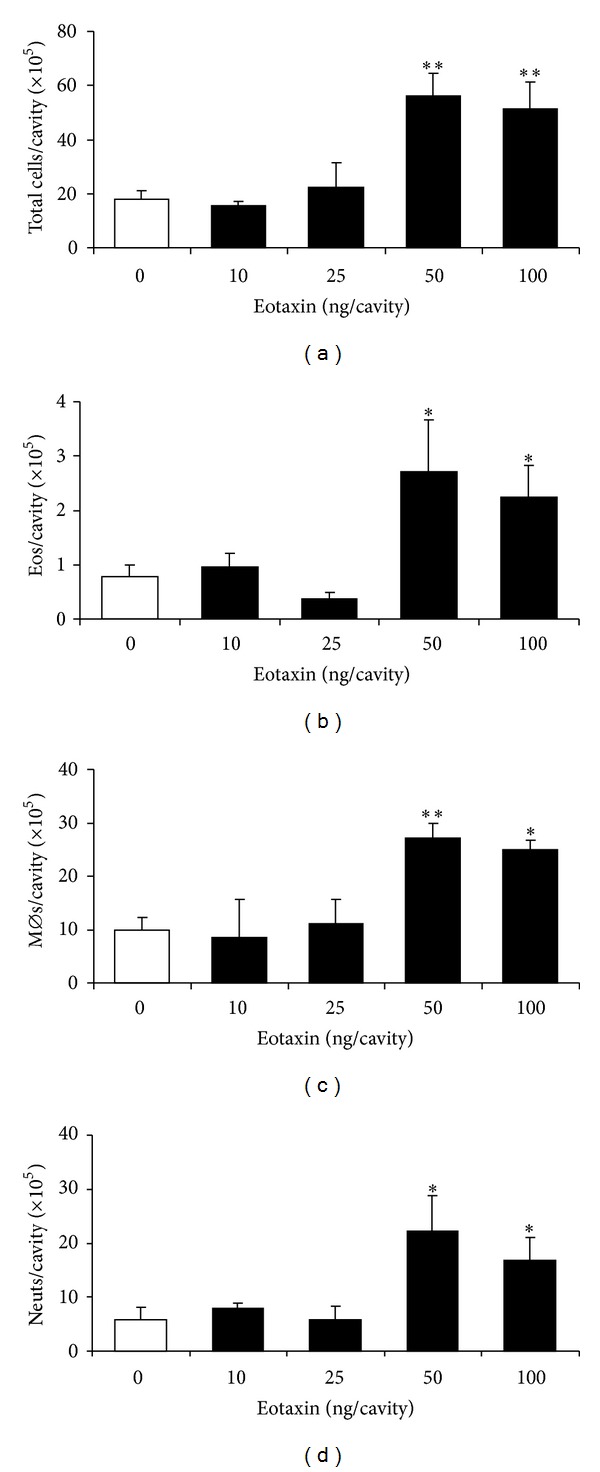
Accumulation of different leukocyte subtypes induced by eotaxin in naive mice: dose-response relationship. BALB/c mice were injected with the indicated doses of eotaxin (black bars), and the peritoneal lavage fluid collected 4 h later was used for quantitation of total leukocytes (a), eosinophils (b), macrophages (c), and neutrophils (d). Data are mean ± SEM. *, *P* ≤ 0,05; **, *P* ≤ 0,01, for the differences relative to the negative (RPMI) control (0 ng/mL eotaxin, open bars). (a) Data from 3–18 experiments. (b)–(d) Data from 6–11 experiments.

We initially examined whether i.p. injection of eotaxin in various doses would recruit eosinophils in a relatively short period (4 h) and whether eosinophil accumulation would be selective, as previously observed in sensitized mice, or accompanied by migration of other leukocyte populations. As shown in Figure 1(a), leukocytes accumulated in response to 50 and 100 ng/cavity eotaxin, in amounts that were significantly different from the RPMI controls (0 ng/cavity) as well as from lower doses of eotaxin (10 and 25 ng/cavity). These leukocytes included variable numbers of eosinophils (Figure 1(b)), monocytes/macrophages [39, 40] (Figure 1(c)), and neutrophils (Figure 1(d)). The morphology of all three leukocyte populations was recognizable without ambiguity, as shown in a representative photomicrograph (supplementary Figure 1 available online at http://dx.doi.org/10.1155/2014/102160). Lymphocyte and basophil migration was not significant in any of these doses (not shown). Importantly, neutrophils and macrophages greatly outnumbered eosinophils, with counts, respectively, 8.2- and 9.9-fold greater in the experiment shown. For all three leukocyte populations, the dose-response relationships were identical, and in subsequent experiments 50 ng/mL was used as the standard stimulus, since no improvement was observed at a higher dose.

**Figure 2:**
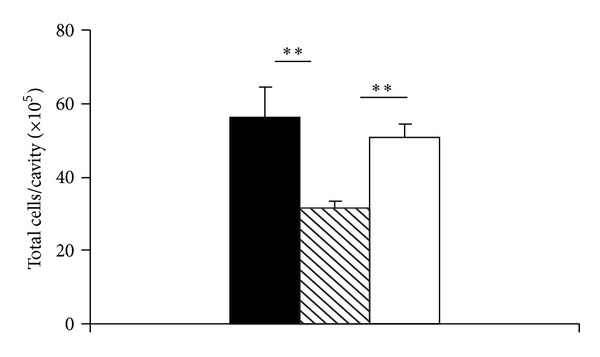
Accumulation of different leukocyte subtypes induced by eotaxin: effect of specific antibody neutralization. Eotaxin was preincubated with specific neutralizing monoclonal antibody (hatched bar), or with irrelevant isotype-matched monoclonal antibody (open bar), before i.p. injection in BALB/c mice. Controls (black bar) received eotaxin but no antibody. Peritoneal lavage fluid, collected 4 h later, was used for total leukocyte quantitation. Data are mean ± SEM. **, *P* ≤ 0,01, for the differences relative to the positive (eotaxin) and specificity (irrelevant antibody) controls. Data from 5–18 experiments.

Despite the heterogeneity of the recruited leukocyte population, neutralization of eotaxin with specific monoclonal antibody brought leukocyte accumulation to negative control levels (Figure 2; compare with Figure 1(a) for the 0–25 ng eotaxin dose range), while control antibody of the same isotype with irrelevant specificity had no effect. This confirms that the stimulus for recruitment of all three leukocyte populations is eotaxin itself, not any unidentified contaminant, which by definition would not be neutralized by specific antibody.

**Figure 3:**
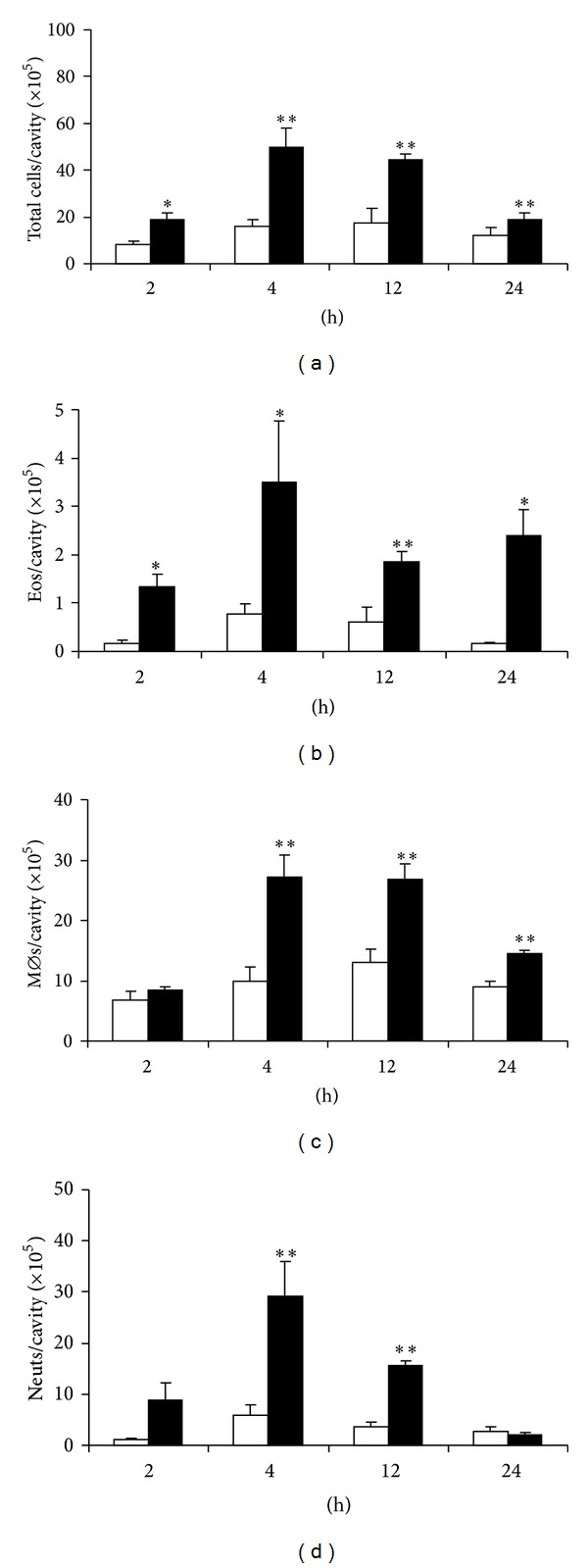
Accumulation of different leukocyte subtypes induced by eotaxin: kinetics. BALB/c mice were injected with 50 ng eotaxin i.p. (black bars), and the peritoneal lavage fluid collected after the indicated periods was used for quantitation of total leukocytes (a), eosinophils (b), macrophages (c), and neutrophils (d). Data are mean ± SEM. *, *P* ≤ 0,05; **, *P* ≤ 0,01, for the differences relative to the respective negative (RPMI) controls (open bars). Data from 3–11 experiments.

The kinetics of recruitment of this mixed leukocyte population by eotaxin in naive BALB/c mice shows significant accumulation as early as 2 h, with a maximum at 4 h, thereafter decreasing but remaining significant at 12 and 24 h (Figure 3(a)). We can observe very early arrival of eosinophils (significant from 2 h and remaining so at 12 and 24 h, Figure 3(b)). By contrast, accumulation of both monocytes/macrophages (Figure 3(c)) and neutrophils (Figure 3(d)) became significant only at 4 h. Significant accumulation was also observed at 12 and 24 h for monocytes/macrophages and 12 h for neutrophils. Hence, monocyte/macrophage and neutrophil accumulation followed eosinophil entry. Eosinophils outlasted neutrophils, but not monocytes/macrophages, in the observation period. For subsequent experiments, the 4 h observation time was chosen, because it showed significant accumulation of eosinophils, monocytes/macrophages, and neutrophils in naive BALB/c mice.

### 3.2. Relationship to 5-LO

**Figure 4:**
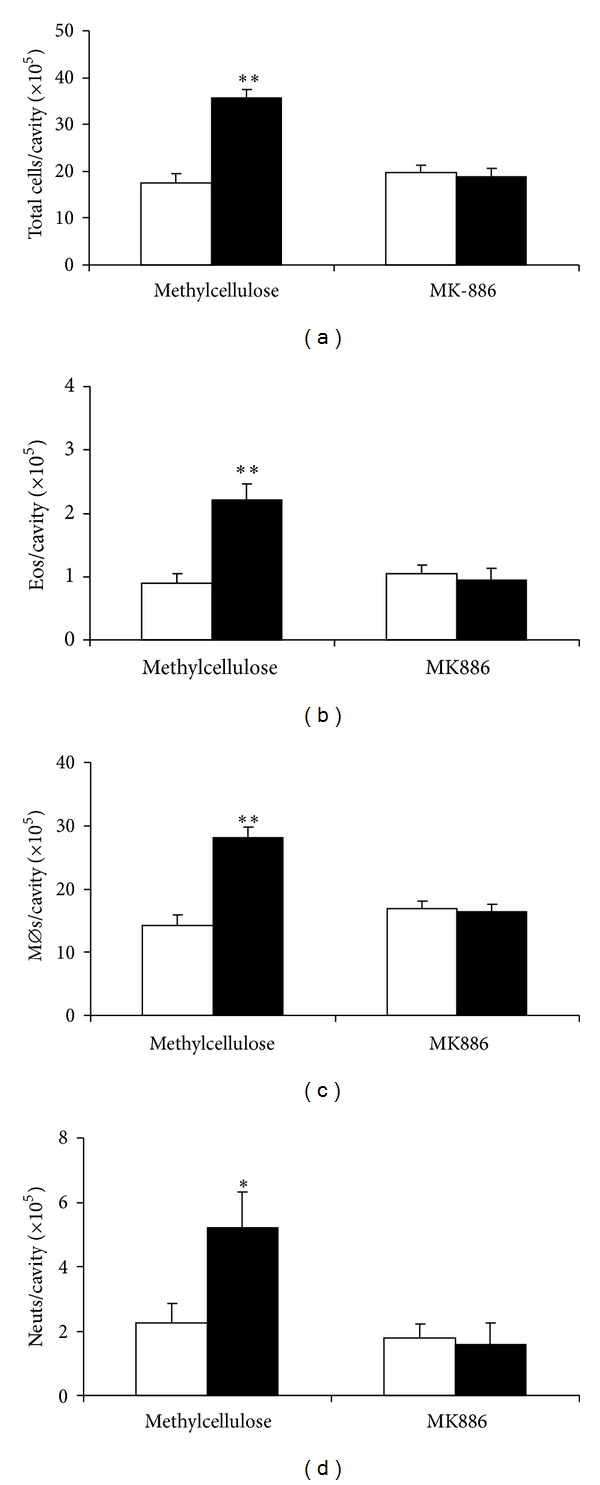
Accumulation of different leukocyte types induced by eotaxin: effect of MK886. BALB/c mice were pretreated with vehicle (methylcellulose) or MK886 and injected with RPMI medium (negative control, open bars) or eotaxin, 50 ng/cavity (black bars). Peritoneal lavage fluid collected after 4 h was used for quantitation of total leukocytes (a), eosinophils (b), macrophages (c), and neutrophils (d). Data are mean ± SEM. *, *P* ≤ 0,05; **, *P* ≤ 0,01, for the differences relative to the respective negative control in each group. Data from 6 experiments.

We first evaluated the effect of eotaxin in naive BALB/c mice pretreated with FLAP inhibitor MK886 or vehicle. MK886 abolished mixed leukocyte recruitment by eotaxin (Figure 4(a)). By contrast, vehicle-pretreated control animals showed significant leukocyte recruitment. MK886 was very effective in preventing eosinophil accumulation (Figure 4(b)). BALB/c mice responded to eotaxin with significant monocyte/macrophage accumulation by 4 h, which was abolished by MK886 (Figure 4(c)). MK886-pretreated BALB/c mice showed no neutrophil migration in response to eotaxin, while migration was significant in vehicle-treated controls (Figure 4(d)).

**Figure 5:**
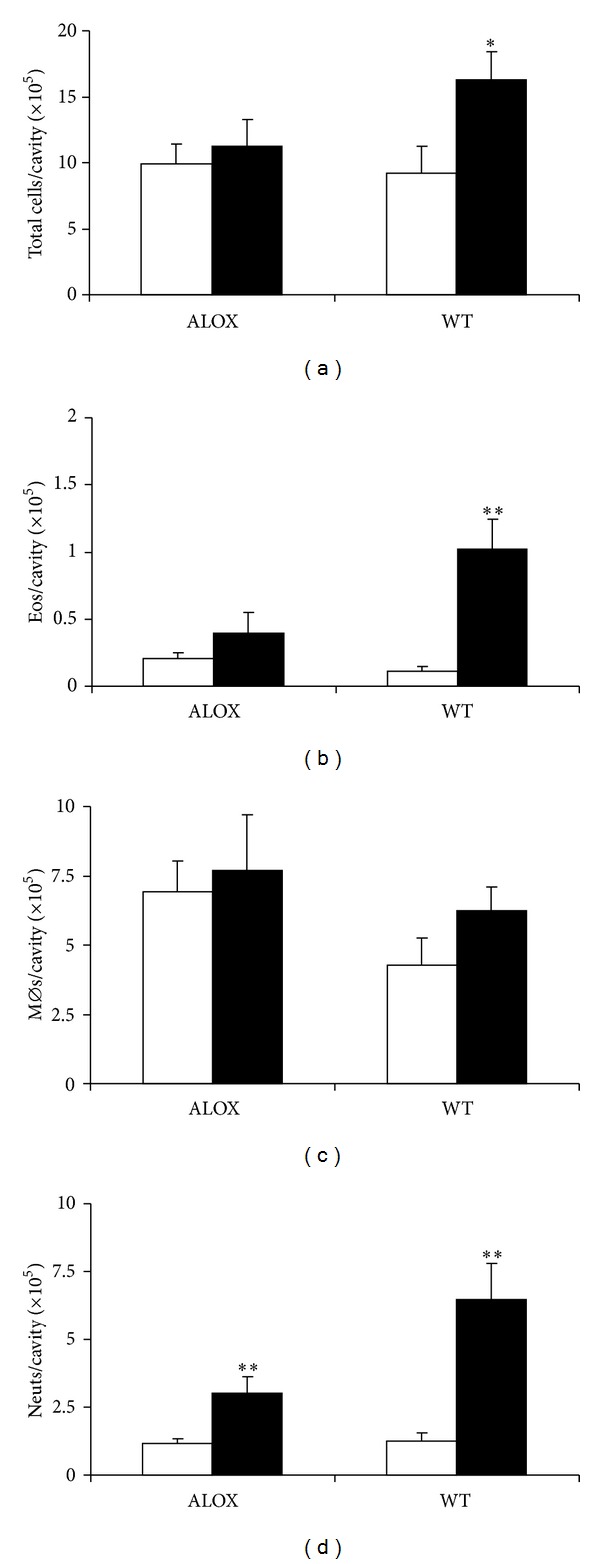
Mixed leukocyte accumulation induced by eotaxin in ALOX and PAS mice. 5-LO-deficient mutant (ALOX) and wild-type (WT) control PAS mice were injected with RPMI (open bars) or eotaxin, 50 ng/cavity (black bars). The peritoneal lavage fluid collected after 4 h was used for quantitation of total leukocytes (a), eosinophils (b), macrophages (c), and neutrophils (d). Data are mean + SEM. *, *P* ≤ 0,05; **, *P* ≤ 0,01. Data from 10 experiments.

Next, we evaluated the effect of eotaxin in naïve ALOX mice, which lack 5-LO, and wild-type PAS controls. In ALOX mice, eotaxin had no significant effect on total leukocyte numbers. By contrast, significant recruitment was observed in PAS controls (Figure 5(a)). Importantly, ALOX mice, unlike PAS controls, showed no significant eosinophil recruitment (Figure 5(b)). In this genetic background, unlike BALB/c, no significant monocyte/macrophage recruitment by eotaxin was observed at this time point (4 h; Figure 5(c)), regardless of whether mice were 5-LO-deficient or wild-type; furthermore, monocyte/macrophage numbers were higher in ALOX than in PAS mice. By contrast, neutrophil recruitment was significant in PAS controls and inhibited by *≈*55% in ALOX mice, although residual neutrophil recruitment remained significant (Figure 5(d)). Together, these observations show that, in this genetic background, eosinophils and neutrophils differ in their requirements for 5-LO to migrate in response to eotaxin, which are total for the former but only partial for the latter.

### 3.3. Eosinophil-Dependent Neutrophil and Monocyte/Macrophage Migration

**Figure 6:**
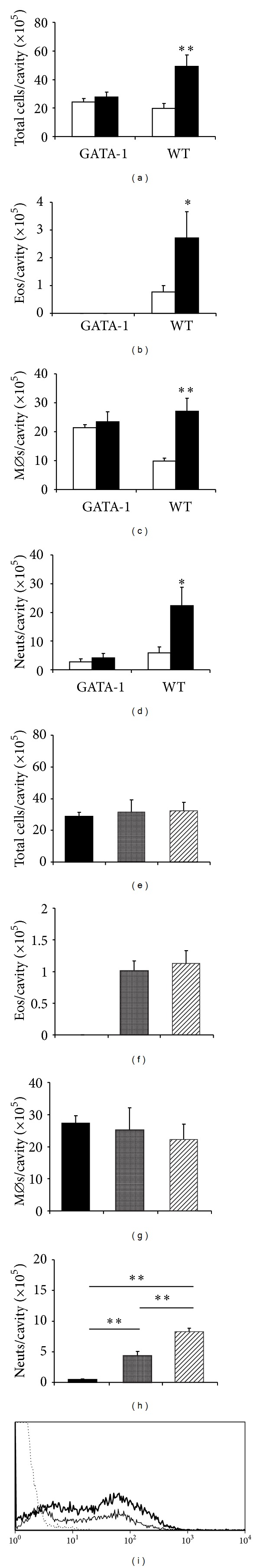
Effect of eosinophil transfer into GATA-1 recipients on neutrophil accumulation. (a)–(d) Eosinophil-deficient GATA-1 mice and wild-type (WT) controls (BALB/c) were injected with RPMI (open bars) or eotaxin, 50 ng/cavity (black bars). The peritoneal lavage fluid collected after 4 h was used for quantitation of total leukocytes (a), eosinophils (b), macrophages (c), and neutrophils (d). E-H, GATA-1 mice received eotaxin (black bars), BALB/c eosinophils (stippled bars), or BALB/c eosinophils followed by eotaxin administration 30 minutes later (hatched bars). Peritoneal lavage fluid collected after 4 h of eotaxin administration was used for quantitation of total leukocytes (e), eosinophils (f), macrophages (g), and neutrophils (h). Data are mean ± SEM. *, *P*≤ 0,05; **, *P*≤ 0,01, for the indicated differences. Data from 3–11 experiments. (i) Intensity of CCR3 expression in granulocytes. Cells were collected 4 h after eotaxin injection from the peritoneal cavity of GATA-1 and BALB/c donors and stained for CCR3. Representative MFI profiles for the granulocyte gate are shown. Dotted line, GATA-1. Thin line, BALB/c. Thick line, GATA-1 sample to which purified BALB/c eosinophils were added in vitro (up to 20% of total cells).

The kinetics of mixed leukocyte recruitment in naive BALB/c mice raised the issue of whether eotaxin-stimulated eosinophils recruit other leukocyte types. If so, neutrophil and/or monocyte/macrophage migration in response to eotaxin would be decreased in the absence of eosinophils. Since naive mice carrying a mutation in the high-affinity GATA-1 binding site of the promoter from the gene coding for the GATA-1 transcription factor lack eosinophils [4], we evaluated the effect of eotaxin on leukocyte numbers 4 h after i.p. injection in GATA-1 mutant mice and BALB/c wild-type controls. In GATA-1 mice, unlike BALB/c controls, leukocyte numbers in the peritoneal cavity were not significantly increased by eotaxin (Figure 6(a)). As expected, eosinophils were undetectable in GATA-1 mice,and effectively recruited by eotaxin in BALB/c controls (Figure 6(b)). In both RPMI-treated and eotaxin-treated GATA-1 mice, monocyte/macrophages (which were the predominant resident leukocyte population) were about twice as numerous as in RPMI-treated BALB/c controls (Figure 6(c)), reaching counts comparable to those in eotaxin-treated BALB/c. Importantly, neutrophil numbers were not significantly increased by eotaxin (Figure 6(d)) in GATA-1 mice, unlike BALB/c controls, suggesting that neutrophil recruitment by eotaxin is eosinophil-dependent. To rule out the possibility that neutrophil migration is somehow defective in this strain, separate control GATA-1 mice were injected with thioglycollate broth, which induces an intense neutrophil accumulation in a 4 h period. GATA-1 and BALB/c mice responded equally well to thioglycollate (not shown), indicating that failure of neutrophil recruitment in GATA-1 mice is a feature of their eotaxin response, not evidence of a general defect in neutrophil migration.

We further explored this issue by reconstituting a peritoneal eosinophil population in GATA-1 mice by transfer of purified (90%) BALB/c eosinophils, devoid of neutrophil contamination. Total leukocyte counts were not significantly different between GATA-1 mice given eotaxin alone, eosinophils alone, or eotaxin plus eosinophils (Figure 6(e)), and this was closely paralleled by monocyte/macrophage counts, which account for most leukocytes in all groups (Figure 6(f)). As expected, eosinophils could be recovered from GATA-1 recipients of eosinophils, and eotaxin did not significantly increase their numbers, as the recipients produce no eosinophils of their own (Figure 6(g)). Importantly, neutrophil numbers were significantly increased by eosinophil transfer and further significantly increased by the association of eosinophil transfer and eotaxin (Figure 6(h)). Together, these data suggest that in naive mice eosinophils mediate the accumulation of neutrophils induced by eotaxin.

If, as suggested by the preceding results, neutrophils and monocyte/macrophages accumulate in GATA-1 mice as a result of eosinophil activation, not of direct exposure to eotaxin, one should expect the leukocytes harvested from the peritoneal cavity of GATA-1 mice to show little or no expression of CCR3, unlike eosinophils. We have therefore compared the expression of CCR3 in peritoneal lavage leukocytes from BALB/c and GATA-1 mice collected 4 h after eotaxin injection (Figure 6(i)). Mean fluorescence intensity was monitored in the granulocyte region, since our transfer protocol reconstitutes migration of neutrophils, not monocytes/macrophages (see above). No eotaxin-induced recruitment of CCR3+ granulocytes was observed in GATA-1 mice (dotted line), unlike BALB/c mice (thin line). To make sure that CCR3+ cells would be detectable, if present in a suspension of GATA-1 granulocytes, we also added purified BALB/c eosinophils to GATA-1 leukocytes as a control (Figure 6(i), thick line). Exogenously added CCR3+ cells were easily detectable in these conditions.

**Figure 7:**
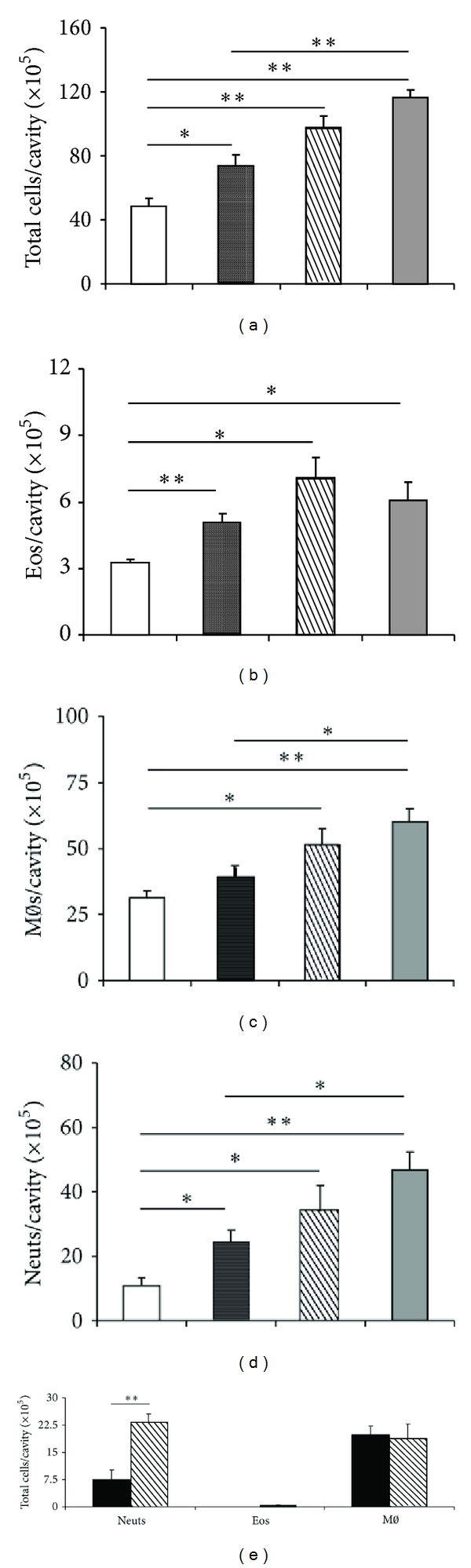
Effect of transfer of PAS or ALOX eosinophils on neutrophil accumulation in the peritoneal cavity of ALOX, PAS, and GATA-1 recipients. (a)–(d) ALOX mice received RPMI (open bars), PAS eosinophils (stippled bars), PAS eosinophils followed by eotaxin, 50 ng/cavity, 30 minutes later (hatched bars). As positive controls, PAS mice received PAS eosinophils followed by eotaxin (gray bars). Peritoneal lavage fluid collected 4 h after eotaxin injection was used for quantitation of total leukocytes (a), eosinophils (b), macrophages (c), and neutrophils (d). (e) GATA-1 mice received eotaxin (black bars), or ALOX eosinophils, followed by eotaxin, 30 minutes later (hatched bars). Peritoneal lavage fluid collected 4 h after eotaxin administration was used for quantitation of neutrophils (Neuts), eosinophils (Eos), and macrophages (M*ϕ*). Data are mean ± SEM. *, *P* ≤ 0,05; **, *P* ≤ 0,01, for the indicated differences. Data from 3–5 experiments.

We took advantage of the effectiveness of eosinophil transfer to examine the relationship of 5-LO to the migration of eosinophils, as well as to the secondary recruitment of neutrophils and monocytes/macrophages. A mixed leukocyte population accumulated in the peritoneal cavity of ALOX recipients of PAS eosinophils (Figure 7(a)), 4 h following administration of eotaxin. No significant improvement was observed in PAS recipients of PAS eosinophils in the same conditions, showing that recruitment is as effective in the ALOX recipients as in the wild-type recipients. The recruited leukocyte population from ALOX recipients included eosinophils (Figure 7(b)), comprising both the transferred eosinophils and those recruited by eotaxin administration to the recipients, again reaching levels comparable to those of PAS recipients of PAS eosinophils. Secondary recruitment was observed for both macrophages (Figure 7(c)) and neutrophils (Figure 7(d)), with similar effectiveness in comparison to the PAS into PAS transfers.

We next examined whether the critical step requiring 5-LO in this model is the initial eosinophil accumulation, rather than the secondary recruitment of neutrophils by eosinophils. If so, one would predict that direct transfer of ALOX eosinophils into eosinophil-deficient GATA-1 recipients should restore neutrophil accumulation in response to eotaxin. When purified eosinophils from ALOX bone-marrow cultures were transferred to GATA-1 recipients (Figure 7(e)), recruitment of neutrophils was very effective. This rules out the possibility that the step critically dependent on 5-LO is the generation by eosinophils of a neutrophil chemoattractant. On the other hand, as shown above for BALB/c eosinophil transfer into GATA-1 recipients, monocytes/macrophages were not increased by ALOX eosinophil transfer at this time point.

### 3.4. Impact on Granulocyte Interaction with Bacteria

**Figure 8:**
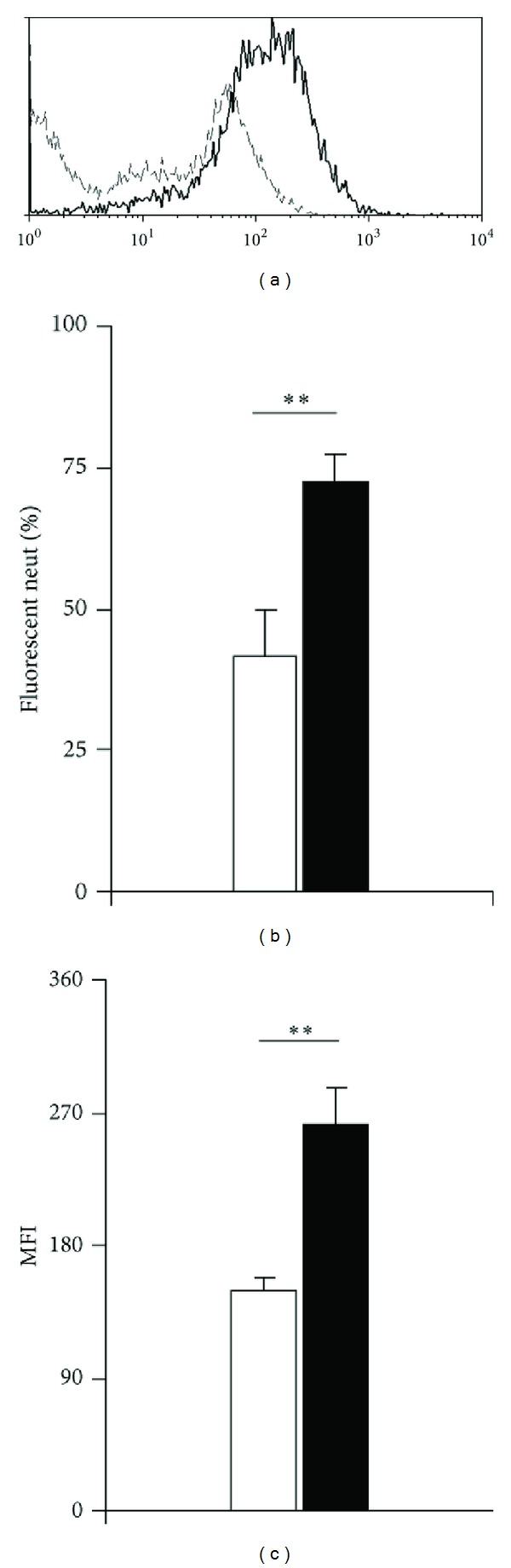
Flow cytometric analyses of peritoneal lavage leukocytes. (a)–(c) Interaction between fluorescent bacteria and neutrophils from eotaxin-injected and control mice. BALB/c mice were injected with eotaxin 50 ng/cavity i.p. Controls received RPMI. After 4 h, peritoneal lavage fluid was collected from both groups. After counting neutrophils, fluorescent, viable *E. coli* bacteria were mixed with leukocytes at a 400 : 1 bacteria/neutrophil ratio and further incubated for 30 min before washing to eliminate unbound bacteria, and analysis of neutrophil-associated fluorescence by flow cytometry. (a) Representative profiles of eotaxin-induced (thick, continuous line) and RPMI-induced (thin, interrupted line) neutrophil-associated fluorescence. (b) Fraction of the neutrophils positive for fluorescent bacteria in RPMI-induced (open bar) and eotaxin-induced (black bar) peritoneal leukocyte populations (mean ± SEM); (c) mean fluorescent intensity of neutrophils in the same samples (mean ± SEM). Data from 4-5 experiments.

We further examined whether eosinophil-mediated responses to eotaxin in this model had an effect on the ability of the secondarily recruited neutrophils and their bacterial targets. To do so, mixed leukocyte populations induced by eotaxin (RPMI in controls) were collected from naive BALB/c mice at 4 h after injection, counted, and mixed for 30 minutes with GFP-expressing *E. coli* at a bacteria/leukocyte ratio adjusted to 400 : 1, before analysis by flow cytometry. Cells gated in the granulocyte region on the basis of size and complexity were examined for green fluorescence, resulting from both binding and internalization of bacteria.  Figure 8(a) shows that eotaxin-stimulated granulocytes bind/internalize fluorescent *E. coli* bacteria more effectively than those collected from RPMI-injected control mice. This increase in effectiveness is detectable as an increased fraction of granulocytes binding bacteria (Figure 8(b)) and an increased mean fluorescent intensity (Figure 8(c)). This suggests that eosinophil-mediated recruitment of neutrophils is accompanied by an increased capacity to bind and/or ingest bacteria.

## 4. Discussion

We describe here a mixed leukocyte accumulation occurring in the peritoneal cavity of naive mice injected with eotaxin. This is, to our knowledge, the first experimental evidence that *recruitment of neutrophils and macrophages by eotaxin in nonsensitized animals is mediated by eosinophils*. For neutrophils, recruitment was associated with an increased ability to bind/ingest bacteria and therefore might have an impact on antimicrobial defenses in specific conditions. Because the ability of eosinophils to act as effective antimicrobial defenses is limited by their scarcity, these findings also highlight conditions in which, by recruiting much larger numbers of cells with well-characterized microbicidal function, eosinophils actually overcome this theoretical disadvantage.

We will below address a number of specific points which are important for putting our observations in a proper perspective.

### 4.1. Roles of Eotaxin, Eotaxin Receptors, and Eosinophils

Migration of all three leukocyte types in BALB/c mice was induced by eotaxin, as shown by identical dose-response relationships and overlapping kinetics, as well as by identical effects of neutralizing eotaxin with specific antibodies. The relationship of this migration to the expression of CCR3, by contrast, is more complex. Lymphocytes, some of which have been shown by others to express CCR3 [41, 42], were not attracted by eotaxin to the peritoneal cavity of naive mice in significant numbers. On the other hand, despite the commonly held view that CCR3 expression is restricted to eosinophils [4, 18, 20, 22, 23], basophils [24, 25], eosinophil and basophil progenitors/precursors [26, 27], T cell subsets [41, 42], and smooth muscle cells [29], several studies have suggested that human and murine neutrophils and macrophages can also express CCR3, at least in specific experimental settings [28, 32], as suggested by studies in neutrophils [43]. This would imply that all three leukocyte populations shown to be recruited in our study in wild-type (BALB/c, PAS) mice could be simply responding to eotaxin binding to CCR3 at the individual cell level, with no contribution from cellular interactions involving eosinophils. If so, there should be no decrease in neutrophil or macrophage accumulation by eliminating eosinophils, but one should expect neutrophils and macrophages to express CCR3 at significant levels even in eosinophil-deficient GATA-1 mice. This possibility, however, has been directly ruled out by the demonstration that eotaxin in GATA-1 mutant mice does not recruit neutrophils nor macrophages. The evidence for cellular interactions in the neutrophil response to eotaxin is reinforced by experiments using the same strain, which show neutrophil recruitment following transfer of highly purified BALB/c eosinophils. Finally, we observed no significant accumulation of CCR3+ granulocytes in eotaxin-injected GATA-1 mice.

By contrast, GATA-1 mice had constitutively increased macrophage numbers in the peritoneal cavity, which were unaffected by 4 h of eotaxin administration, both with and without eosinophil transfer. It is possible that the GATA-1 mutation affects the cellular function, tissue distribution, and/or turnover of monocytes/macrophages so as to prevent responses to eotaxin, regardless of whether these are mediated or not by eosinophils. Therefore, we cannot conclude from our present observations in GATA-1 mice alone that eosinophils also recruit monocytes/macrophages. Direct evidence for eosinophil recruitment of monocytes/macrophages was, however, obtained through transfer of eosinophils from PAS donors into ALOX mice. Importantly, in the absence of eosinophil transfer, ALOX mice showed neither eosinophil nor monocyte/macrophage recruitment by eotaxin. Interestingly, although in the direct stimulation protocol ALOX mice resembled GATA-1 mice, in their absence of monocyte/macrophage accumulation by 4 h, it is likely that different mechanisms underlie these similar outcomes, since (a) a similar failure to respond to eotaxin with monocyte/macrophage accumulation was observed in wild-type PAS controls and cannot therefore be ascribed to the absence of active 5-LO; (b) transfer experiments show that eosinophil transfer from PAS donors allows a significant recruitment of monocytes/macrophages by eotaxin in ALOX recipients. These observations suggest that an active 5-LO is not required for monocytes/macrophages (or neutrophils) to respond to eotaxin, provided eosinophils are present.

Overall, the data indicate that eotaxin recruits a mixed leukocyte population in naive mice through a mechanism dependent on eosinophils. Evidence that eosinophils play an active role is provided by the observation that full effect in eosinophil transfer experiments requires both eosinophils and eotaxin, which would not be expected if eosinophils played a merely passive or permissive role. On the other hand, in transfer experiments about 10% of the transferred eosinophils were recovered by 4 h eotaxin stimulation of the recipients. This raises the issue of whether the remaining transferred eosinophils underwent changes such as degranulation [4] or release of extracellular traps [10], which might represent a significant difference relative to the direct (nontransfer) protocol used in the initial experiments.

### 4.2. Role of 5-LO

Mixed leukocyte recruitment by eotaxin in naive mice shows the same dependence on 5-LO that was observed for selective eosinophil recruitment in sensitized mice. Hence, it is likely that eosinophil accumulation itself, the shared feature in both models, is the 5-LO-dependent step. This is consistent with the observation that ALOX eosinophils, when directly transplanted to the peritoneal cavity of GATA-1 recipients (which are unable to respond to eotaxin by accumulation of neutrophils), are able to mediate the neutrophil recruitment induced by eotaxin. Importantly, further recruitment of eosinophils occurs in eotaxin-stimulated ALOX recipients of PAS eosinophils, where the only cells bearing a functional 5-LO are the transferred eosinophils. This suggests that eosinophils can be a source as well as a target for a 5-LO pathway product, such as LTB4. LTB4 was previously shown to selectively attract eosinophils, in a model in which eotaxin duplicated the effect of antigen in a 5-LO-dependent manner [33]. Furthermore, there is significant evidence that recruitment involves interactions between cytokines and lipid mediators [44]. In neutrophil migration, LTB4 represents a signaling relay, raising the possibility that it acts similarly in eosinophils [45]. Whatever mechanism is involved, eosinophil generation of a 5-LO-derived neutrophil chemoattractant is not required for the eosinophil-dependent secondary recruitment of neutrophils in eotaxin-injected naïve mice. While in previous studies of sensitized mice, LTB4, as did antigen and eotaxin, selectively recruited eosinophils [34], it remains to be determined whether it accounts for the rapid eosinophil recruitment to the peritoneal cavity of the eotaxin-injected nonsensitized mice in the present study. A related issue for further investigation is whether 5-LO is required for the increased effectiveness of bacterial binding that was detected in BALB/c leukocytes, as LTB4 is known to activate as well as attract neutrophils [45].

### 4.3. Relationship to Innate and Acquired Immunity

Despite a common requirement for 5-LO, leukocyte recruitment by eotaxin differs in several aspects between naive versus sensitized mice, especially the lack of eosinophil selectivity in the former, as opposed to the latter. In sensitized mice, selective eosinophil recruitment was observed with widely different chemical stimuli (allergen, eotaxin, or LTB4). It is unlikely, therefore, that such selectivity reflects some features of eotaxin signaling and even less of LTB4 signaling (which should be very effective in mice having normal neutrophil numbers). Alternatively, the failure of eotaxin and LTB4 to recruit neutrophils and monocytes/macrophages in sensitized mice could involve changes in the expression of adhesion proteins at endothelial surfaces, which would prevent their emigration from blood vessels to peritoneal cavity, regardless of whether the chemoattractant is LTB4 or eotaxin. We have not examined this possibility, since our current observations, which are centered on responses from nonsensitized animals, do not depend on clarifying mechanisms that were not applicable to the present conditions.

We view our findings as manifestations of innate immunity, because of (a) the very fast kinetics of eosinophil and neutrophil accumulation; (b) the recruitment of neutrophils and macrophages in the absence of significant lymphocyte accumulation; (c) the detectable increase in granulocyte binding of live extracellular bacteria in the absence of antibodies. On the other hand, the fast recruitment of monocytes/macrophages by eotaxin-exposed eosinophils raises the issue of whether eosinophils could also enhance protection from more specialized pathogens, such as the intracellular mycobacteria and protozoa that cause chronic infections, which are usually handled by monocytes/macrophages. Relatively little attention has been paid to the possibility that eosinophils play a role in fighting microbial pathogens with the help of other leukocyte types. Our observations suggest that small numbers of eosinophils might recruit a large neutrophil and/or macrophage infiltrate. *While this would make eosinophils surprisingly effective players in innate immunity, this might paradoxically obscure their contribution, if their contribution were to be taken as commensurate with their numbers in inflammatory infiltrates, where they would often amount to no more than one-tenth of total leukocytes*. It is therefore fortunate that, in transfer experiments of wild-type and mutant eosinophils into eosinophil-null GATA-1 mice, eosinophil recruitment of neutrophils can be unequivocally demonstrated. In view of the differences between naive and sensitized models in this respect, it is of interest to determine whether this eosinophil functional capacity is modified by allergen sensitization of the host and whether such a change in innate immune functions can be duplicated by passively or actively sensitizing the host.

### 4.4. Possible Cellular Mechanisms Underlying the Effect of Eosinophils

Several, but not all, of the observations reported here are consistent with those of previous studies, carried out by other groups in different experimental models. Eotaxin recruitment of a mixed leukocyte population, including neutrophils and macrophages, was described in human subjects [28]; Das and colleagues [46] reported that eotaxin was effective when injected in the peritoneal cavity of mice but not in a dorsal air pouch, drawing attention to the important differences between challenge sites responding to the same chemically defined stimulus. Responses in the air pouch occurred after local inoculation of mast cell-containing peritoneal cell populations, but allergen sensitization was essential to local responses to eotaxin in this transfer model. In addition, neutrophil migration accompanied recruitment of eosinophils in specific conditions. Harris and colleagues [47] confirmed that mast cells were important for full responses to eotaxin and further showed that eotaxin responses were blocked by 5-LO inhibitors. Together, these studies suggest that eotaxin effectiveness is constrained in vivo by several factors that may be absent from in vitro (e. g., migration chamber or flow cytometric) studies. These constraints include mast cells and 5-LO. None of these published studies, however, evaluated the contribution of the recruited eosinophils themselves.

We suggest that a cytokine, rather than a 5-LO derivative, is released by eosinophils in the peritoneal cavity, once they have been recruited by eotaxin in the presence of an active 5-LO, or, alternatively, directly inoculated in the cavity through a transfer protocol. Candidate cytokines would include TNF-*α* and TGF-*β*1, both potent neutrophil chemoattractants. One hypothesis that could reconcile our observations with those of Das and Harris and their colleagues [46, 47] would involve the amplification of the role of eosinophils through interactions with resident peritoneal mast cells, since mast cells are an important source of neutrophil chemoattractants, including TNF-*α* [48].

## Supplementary Material

Supplementary Figure: Representative image of the mixed leukocyte infiltrates collected from BALB/c mice, 4 h after i. p. injection of 50 ng eotaxin, and stained with Giemsa. In the center, one eosinophil, recognizable by its orange-stained cytoplasm and donut-shaped nucleus, without segmentation. The field contains three neutrophils, recognizable by pale staining of the cytoplasm and by clearly segmented nuclei, ranging from strongly stained, condensed chromatin, to pyknosis. There is one recognizable lymphocyte at the center. The field contains numerous cells with monocyte/macrophage morphology, which are larger than granulocytes, show clearly-stained cytoplasm, oval or round nuclei with loosely condensed chromatin. In the same page, please note that Figure 6(i) has been modified in a way that makes it difficult for the reader to identify which or the two continuous lines shown is thick, which is thin, as they appear very similar. I would suggest that you refer to the files sent you to make sure you, in which the thick line is the higher continuous line, and there is no confusion possible, and try to depict these two continuous lines in a more distinctive way, to avoid confusing the readers.
