# Supplementary figures and images for: 5-Lipoxygenase-Dependent Recruitment of Neutrophils and Macrophages by Eotaxin-Stimulated Murine Eosinophils
Source: Mediators Inflamm. 2014 Feb 25;2014:102160. doi: 10.1155/2014/102160 (PMC3955596; doi:10.1155/2014/102160)

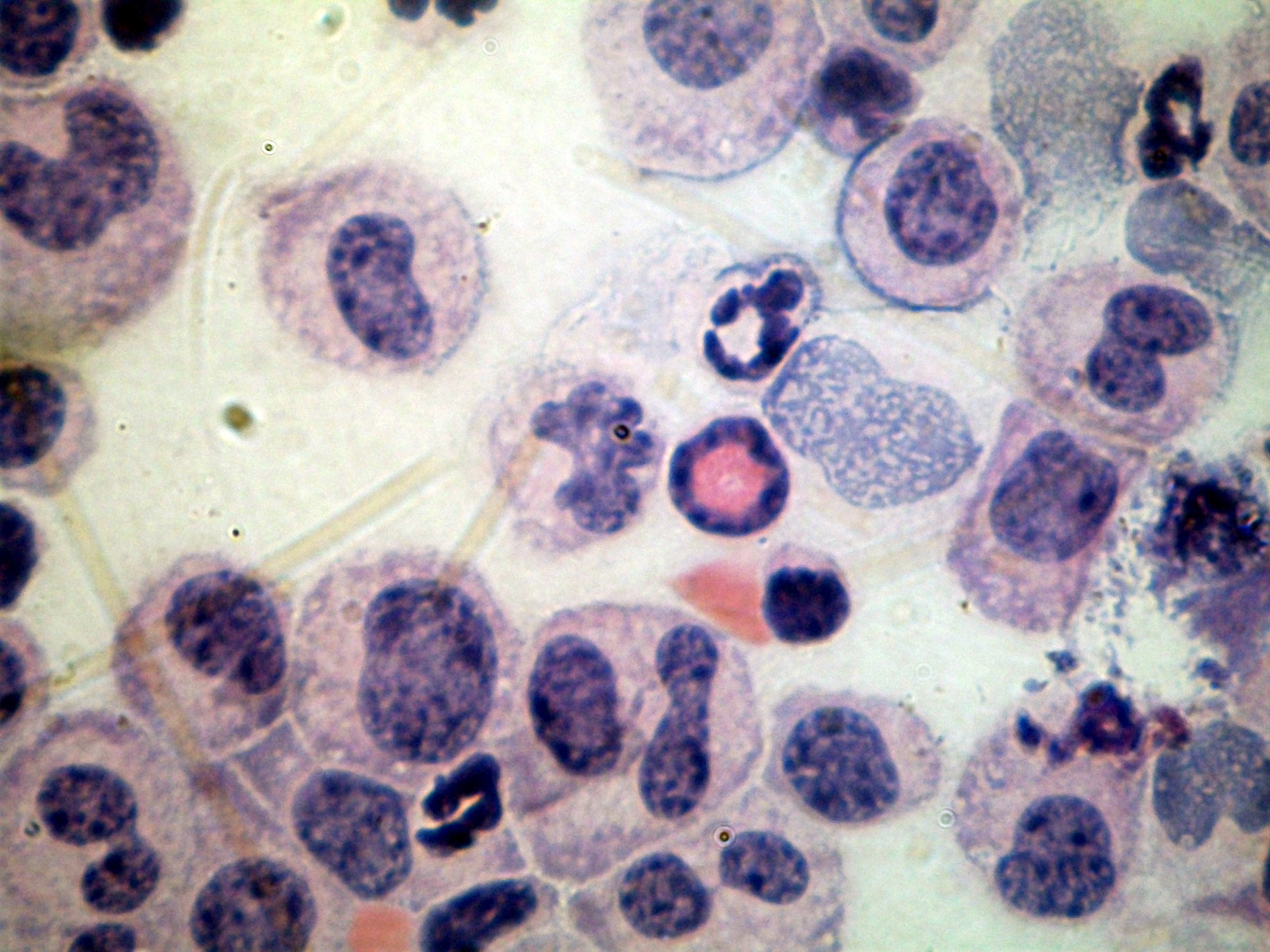

Supplement: Supplementary file 1 — Supplementary Figure: Representative image of the mixed leukocyte infiltrates collected from BALB/c mice, 4 h after i. p. injection of 50 ng eotaxin, and stained with Giemsa. In the center, one eosinophil, recognizable by its orange-stained cytoplasm and donut-shaped nucleus, without segmentation. The field contains three neutrophils, recognizable by pale staining of the cytoplasm and by clearly segmented nuclei, ranging from strongly stained, condensed chromatin, to pyknosis. There is one recognizable lymphocyte at the center. The field contains numerous cells with monocyte/macrophage morphology, which are larger than granulocytes, show clearly-stained cytoplasm, oval or round nuclei with loosely condensed chromatin. In the same page, please note that Figure 6(i) has been modified in a way that makes it difficult for the reader to identify which or the two continuous lines shown is thick, which is thin, as they appear very similar. I would suggest that you refer to the files sent you to make sure you, in which the thick line is the higher continuous line, and there is no confusion possible, and try to depict these two continuous lines in a more distinctive way, to avoid confusing the readers. [file 102160.f1.jpg]
